# High-Quality Natural Fibers from Cotton Stalk Bark via Limited Alkali Penetration and Simultaneous Accelerated Temperature Rise

**DOI:** 10.3390/ma15020422

**Published:** 2022-01-06

**Authors:** Zhen Dong, Na Li, Teye Chu, Jiangxin Ding, Junxiong Zhang, Aixue Dong

**Affiliations:** 1 College of Textiles & Apparel, Nantong University, 9 Seyuan Road, Nantong 226019, China; 2012320001@stmail.ntu.edu.cn (N.L.); 2112320002@stmail.ntu.edu.cn (J.D.); zhangjunxiong@ntu.edu.cn (J.Z.); 2 Jiangxi Enda Technology Co., Ltd., Xinyu 336600, China; chuteye@163.com; 3 Key Laboratory of Cleaning Dyeing and Finishing Technology of Zhejiang Province, Shaoxing University, Shaoxing 312000, China; aixue_dong@126.com

**Keywords:** lignocellulose, agricultural wastes, cotton stalks, natural fibers, lignin, cellulose

## Abstract

High-quality cotton stalk fibers that are both fine and have a high breakage strength are extracted via limited alkali penetration in the glycerol solvent and simultaneous accelerated temperature rise by means of microwave-assisted heating. Alkali is widely used in the extraction of cotton stalk fibers. However, alkali molecules in the aqueous phase penetrate easily into the fiber bundles, resulting in a simultaneous degumming between the inner and outer layers of the fiber bundles. In previous reports, the fibers treated in the aqueous phase present a coarse fineness (51.0 dtex) under mild conditions or have a poor breakage strength (2.0 cN/dtex) at elevated temperatures. In this study, glycerol is chosen as a solvent to reduce the penetration of alkali. Simultaneously, the microwave-assisted heating form is adopted to increase the temperature to 170 °C within 22 s. The inhibited alkali penetration and accelerated temperature rise limited the delignification to the outer layer, resulting in fibers with both appropriate fineness (23.8 dtex) and high breakage strength (4.4 cN/dtex). Moreover, the fibers also exhibit a clean surface and large contact angle. In this paper, we detail a new strategy to extract high-quality lignocellulosic fibers that will be suitable for potential reinforcing applications.

## 1. Introduction

Cotton stalks are one of the major agricultural byproducts that are copious and inexpensive but have limited use in high-value applications. It is reported that about 115 million tons of cotton stalks are generated per year in China [1], most of which are thrown away or burned in the field [2]. The bark of cotton stalks contains about 19.3% hemicellulose, 23.1% lignin and 32.1% cellulose [3]. Extracting high-quality natural fibers from the bark will not only increase the income generated by cotton cultivation but also improve the sustainability of the natural fiber supply [4,5,6].

Alkaline treatment methods are widely used in extracting natural fibers from the agricultural wastes, including thespesia populnea bark [7], grape cane [8], Juncus effusus [9], cotton stalks [10], etc. Most of these alkaline treatments are carried out in the water phase, which is beneficial to the dissolution of lignin and other gum substances. It is generally believed that removing these gum substances may improve the fiber fineness but easily causes a reduction in the fiber strength. Reddy and Yang found that the cotton stalk fibers that were treated in an 8 wt % alkaline aqueous solution at 90 °C had a fineness of 51 dtex [11], too coarse for high valuable applications [12]. When combined with steam explosion and peroxide treatments, alkali treatment might improve the fineness to 27.0 dtex but reduce the fiber strength to 2.2 cN/dtex [3]. In our previous studies [12], the fibers treated at 130 °C in 8 wt % alkaline aqueous solution had the best fineness of 24 dtex but the strength was reduced to only 2.0 cN/dex. To the best of our knowledge, no reports have been available in solving the problem of strength loss during the fiber extraction process.

Controlled delignification is a key to minimize the strength loss of lignocellulose fibers during the fiber extraction process. Lignin macromolecules usually have complex reticulate structures [13,14], which render them more difficult to be degraded than polysaccharides. Moreover, the intramolecular and intermolecular ether bonds are not easily broken under mild conditions [15]. Therefore, only small amounts of lignin in the lignocellulosic fibers can be dissolved at 100 °C or lower temperatures [16]. As the temperature increases to 150 °C or higher, the lignin macromolecules are degraded efficiently. However, the penetration of alkali in the lignocellulosic fibers is accelerated at the same time. The two factors work together, leading to a simultaneous removal of gum between the inner and outer layer of the fiber bundles. As a result, alkali in the aqueous phase might create fine fibers, but usually with poor breakage strength. If the removal of gum can be limited to the outer layer, the loss of fiber strength will be minimized. 

This paper aims to study a novel strategy to extract natural fibers from the bark of cotton stalks with both appropriate fineness and high breakage strength. In this study, glycerol is used as a solvent to reduce the penetration of alkali along the radial direction of the fiber bundles. Meanwhile, the conventional water bath heating is replaced by microwave-assisted heating to speed up the removal of gum. The main experimental parameters including alkali concentration, microwave power and microwave time are optimized for extracting fibers with both appropriate fineness and high breakage strength. The fluorescence microscope images of the fibers cross section are used to observe the penetration of alkali in the cotton stalk fibers. Changes in the composition and inner structure of the cotton stalk fibers are determined. Moreover, the wettability and surface characteristics of the cotton stalk fibers are also analyzed. 

## 2. Materials and Methods

### 2.1. Materials

The bark from cotton stalks was purchased from a farm in Salt City, Jiangsu, China. Alkalophilic bacillus NTT33-Y6 was provided by Jiangxi Enda Technology Co., Ltd. (Jiangxi, China). Sodium hydroxide, glycerol and other chemicals in this study were reagent-grade and supplied by Nantong Instrument Co. Ltd., Nantong, China. 

### 2.2. Methods

#### 2.2.1. Preparation of Coarse Fiber Bundles

Coarse fiber bundles were separated from the cotton stalk bark using alkalophilic bacillus NTT33-Y6 according to the method described in our previous studies [17]. About 150 g of cotton stalk bark was sterilized at 120 °C and then added to a fermentation tank containing 4 g of Na_2_CO_3_, 2.5 g of (NH_4_)_2_SO_4_, 1.2 g of K_2_HSO_4_ and 1.2 kg of water. Afterwards, the bacillus NTT33-Y6 was inoculated. After fermentation at 40 °C for 7 h, the coarse fiber bundles were rinsed with water and dried at room temperature. Finally, the coarse fiber bundles were opened using a FN300A model carding machine (Qingdao Jingjia Co. Ltd., Shandong, China).

#### 2.2.2. Extraction of Cotton Stalk Fibers

The opened fiber bundles in Section 2.2.1 (hereafter called the fibers untreated) were coated with NaOH/glycerol mixtures using a liquid to fiber weight ratio of 2:1, heated in a XH200A model microwave heater (Beijing Xianghu Technology Development Co., LTD, Beijing, China), and then rinsed thoroughly with clean water. The main experimental parameters are listed in Table 1, in which the microwave power and time were varied to obtain fibers with both appropriate fineness and high breakage strength. Afterwards, the fibers were washed and dried at room temperature. 

For comparison, the fiber bundles were also treated by NaOH in an aqueous solution. According to our previous studies, temperatures above 150 °C would easily cause a disintegration of the fiber bundles [12]. In this study, we adopted an optimized process parameter, including an 8 wt % alkali concentration at 130 °C for 3 h with a liquid to fiber ratio of 30:1 [12], to obtain fibers with the best fineness of 24.0 dtex. 

#### 2.2.3. Determination of the Viscosity and Temperature of Alkali/Glycerin Mixtures

The viscosity of the alkali/glycerin mixtures was determined using an NDJ-5S model digital rotary viscometer (Shanghai Hengping Scientific Instruments Co., Ltd., Shanghai, China). The temperature of the alkali/glycerin mixtures was detected in real time using an infrared thermometer inside the microwave heater. The data were tested three times and given in the form of mean ± standard deviation.

#### 2.2.4. Determination of the Fiber Dimension

The fineness of cotton stalk fibers was determined according to the method of ASTM D1577-92. The dtex value, a unit of fiber fineness, is defined as grams of the fibers per 10,000 m in length. About three hundred fibers were measured to determine the average length according to the standard of ASTM D5103-07. All data were tested three times and given as mean ± standard deviation.

#### 2.2.5. Determination of the Mechanical Properties of Cotton Stalk Fibers

The tensile strength at break and Young’s modulus of the cotton stalk fibers were determined according to ASTM D 3822-07, and the stress–strain curves were recorded. The fibers were conditioned at 20 °C and relative humidity of 65% for about 24 h, and then tested on a 3385H model universal test instrument (Instron Inc., Norwood, MA, USA). A gauge length of 10 mm and crosshead speed of 10 mm/min were selected. About one hundred fibers were tested for all the data and the mean ± standard deviation was given. 

#### 2.2.6. Characterization of Alkali Penetration in the Cotton Stalk Fibers

In this study, the penetration of alkali into the fiber bundles was characterized using the fluorescence microscope images. According to our previous studies [18], bright green fluorescence area in the images indicates the presence of high-concentration lignin. In this study, changes in the concentration of lignin in the secondary wall were used as an indicator of the alkali penetration. The more remarkable the changes in the brightness of the walls, the more sufficient the alkali penetration. In this study, the penetration depth is recorded according to the extent of the bright green area that shrinks. About 60 fibers were tested for all the data and the mean ± standard deviation was given.

Before the observation of the cross section, the fiber bundles were cut into 10 µm thick slices using a CM1520 model freezing microtome (Leica, Wetzlar, Germany). Afterwards, the cell walls and the intercellular layers were observed using an Axio Vert A1 model inverted fluorescence microscope (Zeiss, Oberkochen, Germany) with a magnification of 630 at the wavelength of 488 nm according to the report of Dong et al. [18]. 

#### 2.2.7. Determination of Fiber Composition

The proportion of lignin, hemicellulose and cellulose in the cotton stalk fibers was determined according to GB5889-86 (China National Standard). Firstly, the fibers were immersed in a mixture of benzene and ethanol to remove the wax. Then, the fibers were treated in an ammonium oxalate solution of 5 g/L at 100 °C to remove the pectin substances. Subsequently, the fibers were treated with 20 g/L NaOH at 100 °C for 3.5 h to determine the content of hemicellulose. Finally, the lignin content was determined using 72 wt % sulfuric acid at room temperature. The content of cellulose is the result of subtracting the wt % of pectin, hemicellulose and lignin from 100%.

#### 2.2.8. Crystalline Structure of Cotton Stalk Fibers

The crystalline structure of the fibers was examined on an X-ray diffractometer (D8-Advance, Bruker Co., Billerica, MA, United States) using a CuKα radiation source (λ = 0.154 nm) with a step size of 0.04° and 2θ angle ranging from 5 to 40°. Blank run data were collected and subtracted from the sample patterns. The crystallinity index (CI) was calculated using Equation (1) according to the report of French and Santiago Cintro’n [19].

(1) $$\mathrm{CI}=\frac{\left(I_{200}-I_{\mathrm{am}}\right)}{I_{200}}\times 100\%$$

where I_200_ is the peak intensity at 2θ angle of about 22.6° and I_am_ is the intensity of the peak at 2θ angle around 18°.

#### 2.2.9. Thermogravimetric Analysis of Cotton Stalk Fibers

Derivative thermogravimetry (DTG) curves of the cotton stalk fibers were recorded on a simultaneous thermal analyzer (STA449F5, NETZSCH-Gerätebau GmbH, Selb, Germany). About 10 mg of fiber powders were heated from 40 to 800 °C under a nitrogen atmosphere at a heating rate of 10 °C/min. 

#### 2.2.10. Wettability of the Cotton Stalk Fibers

The water contact angle of the cotton stalk fibers was measured on an OCA15EC model contact angle apparatus (Data Physics Instruments, Filderstadt, Germany). The data were tested three times and the average value was given.

#### 2.2.11. Morphological Analysis of the Cotton Stalk Fibers

Cotton stalk fibers were sputtering coated with Au at 9 mA current for 120 s and then observed using a Gemini 300 model scanning electron microscope (ZEISS, Oberkochen, Germany) under a voltage of 6 kV. 

#### 2.2.12. FTIR-ATR Spectrum of Cotton Stalk Fibers

FTIR-ATR spectra were recorded on a TENSOR 27 model Fourier Transform Infrared spectrometer (Bruker, Billerica, MA, United States) to identify the hemicellulose and lignin compositions. The wavelength range was from 650 to 4000 cm^−1^ with a resolution of 2 cm^−1^ and an accumulation of 64 scans per sample.

## 3. Results

### 3.1. Effect of Microwave Parameters on the Solution Viscosity

**Figure 1:**
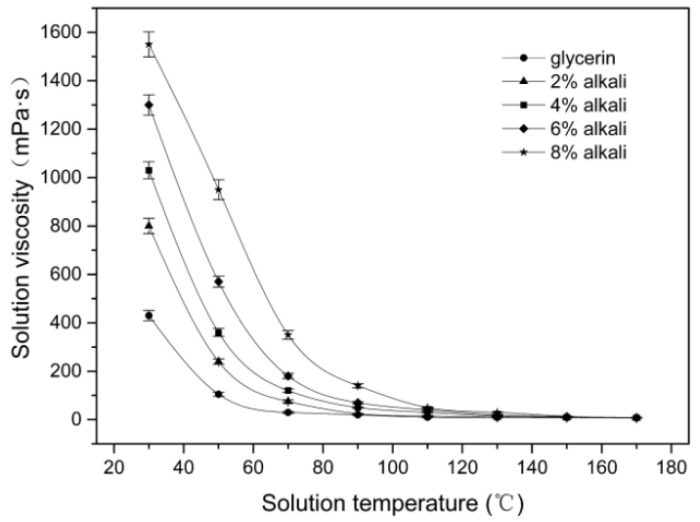
Relationship between the viscosity and temperature of the alkali/glycerin mixtures.

Figure 1 shows the relationship between the viscosity and temperature of the alkali/glycerin mixtures. As seen in Figure 1, the viscosity of alkali/glycerin mixtures shows a gradual decrease with an increase in the temperature. As the temperature rises to about 170 °C, all mixtures have viscosity reduced to a low value of 10 mPa•S. This value is close to the viscosity value (1.03 mPa•s) of water at room temperature. This phenomenon suggests that the alkali/glycerin mixtures will easily penetrate into the fibers at 170 °C. At any given temperature shown in Figure 1, the mixtures with a higher alkali concentration always exhibit a higher viscosity. This result indicates that a high alkali concentration is preferable to a low concentration to inhibit the penetration of alkali. Taking into account the solubility of NaOH in the glycerol solvent, a concentration of 8 wt % is chosen in the subsequent experiments.

**Figure 2:**
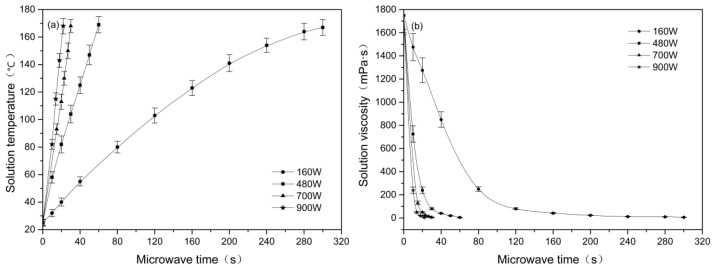
Effect of microwave power and time on the (**a**) temperature and (**b**) viscosity of the alkali/glycerin solutions. All solutions have an alkali concentration of 8 wt %.

Figure 2 shows the effect of the microwave parameters on the temperature and viscosity of the alkali/glycerin solutions. On the whole, the solution temperature rises with an increase in the microwave treatment time. As seen in Figure 2a, it takes 300 s at power of 160 W but requires only 22 s at 900 W to increase the temperature from 25 to 170 °C. Since the time required for raising the temperature is greatly reduced, high power conditions might result in an efficient stripping of single cells. 

As seen in Figure 2b, increasing the microwave time causes a reduction in the solution viscosity. The greater the microwave power, the faster the solution viscosity drops. At a microwave power of 900 W, it takes only 22 s to reduce the solution viscosity from 1750 to 10 mPa•s. However, the corresponding time at 160 W is up to 300 s. From the perspective of inhibiting alkali penetration, the high power condition is unfavorable since the solution viscosity drops too fast.

### 3.2. Effect of Microwave Parameters on the Dimension and Tensile Properties of Cotton Stalk Fibers

**Figure 3:**
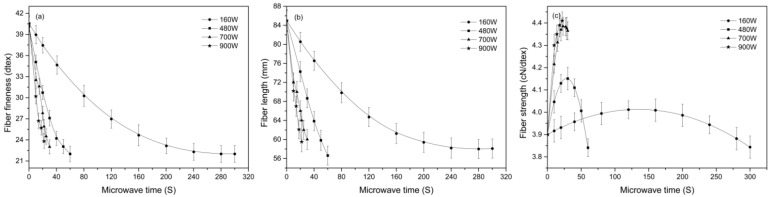
Effect of microwave power and time on the (**a**) fineness, (**b**) length and (**c**) tensile strength at break of the cotton stalk fibers.

Figure 3a,b show the fineness and length of the cotton stalk fibers treated under different microwave conditions, respectively. As seen in Figure 3a, increasing the microwave time causes a decrease in the dtex value. The greater the microwave power, the faster the dtex value drops. In this study, increasing the power speeds up the temperature rise, which will promote the stripping of single cells. Therefore, high power conditions usually accompany with a significant decrease in the dtex value. However, no significant differences are found in the optimum fineness value between the samples under different powers. As seen from Figure 3a, the optimum dtex value varies in a small range from 23.0 to 23.8 dtex when the power increases from 160 W to 900 W. At each microwave power, the fiber extraction process is stopped as the temperature rises to about 170 °C. At such a high temperature, the alkali penetration is fast, and the fiber bundles are easily disintegrated. From this point of view, high power conditions do not bring significant advantages in terms of the optimum fineness value. Compared to the best fineness of 24.0 dtex reported using an alkaline aqueous solution [12], the fibers in this study have the optimum fineness value slightly improved. This result indicates that the high-temperature environment in this study is effective for the stripping of single cells and the improvement of fiber fineness. 

**Figure 4:**
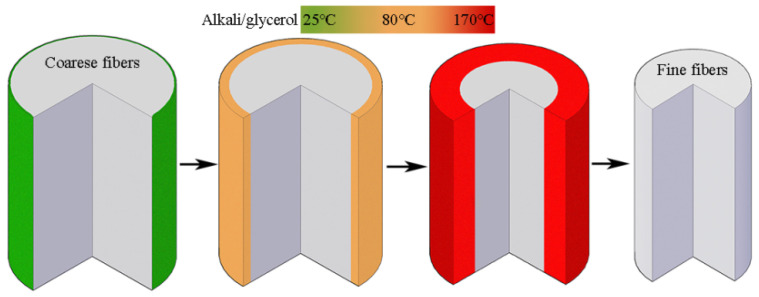
Schematic diagram of the limited alkali penetration in cotton stalk fibers.

Figure 3c shows the tensile strength at break of the fibers treated with different microwave parameters. As seen in Figure 3c, the strength at 160, 480 and 700 W increases first and then decreases gradually with the increase in microwave time, whereas the strength at 900 W shows a unilateral upward trend. According to the report in literature [20], improvement in the fiber diameter and/or the fibrils orientation is responsible for the increase in the fiber strength. In this study, the strength of cotton stalk fibers is influenced by both the rate of the temperature rise and alkali penetration. Under a low microwave power such as 160 W, it takes a long time for the temperature to rise from 25 to 170 °C. Before the solvent temperature reaches 170 °C, the core of fiber bundles has been saturated with alkali. In this case, the fiber strength starts to decrease at the moment alkali reaches the core. Under a high power of 900 W, the alkali/glycerin mixtures are heated up rather quickly. When the temperature reaches 170 °C, alkali molecules have not reached the core yet. In such circumstances, the inner part of the fiber bundles remains intact after the microwave treatment (seen in Figure 4). The model in Figure 4 can be verified from many aspects, including the mechanical properties, composition, and structure of the cotton stalk fibers, as well as the penetration depth of the solvents. 

**Figure 5:**
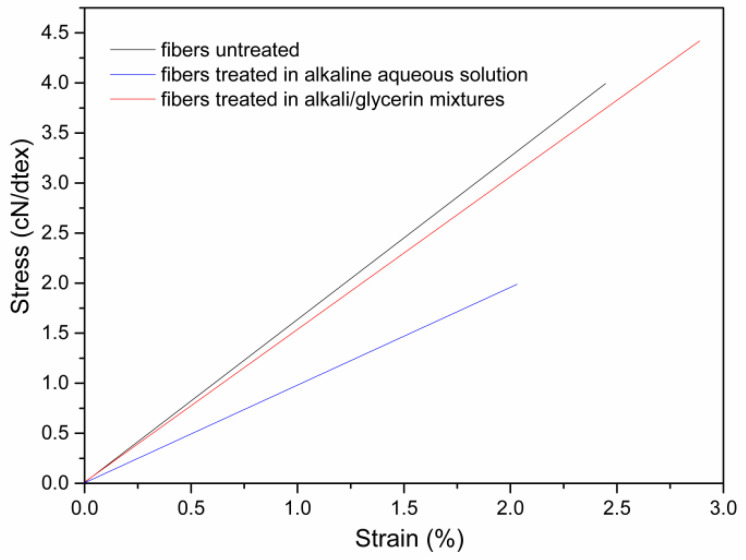
Stress–strain curves of the fibers treated in the alkali/glycerin mixtures compared with those of the fibers treated in the alkaline aqueous solution.

Figure 5 shows the stress–strain curves of the fibers treated in the alkali/glycerin mixtures compared with those of the fibers treated in the alkaline aqueous solution. As seen in Figure 5, the fibers obtained in this study have a high breakage strength of 4.4 cN/dtex and a Young’s modulus of 162.2 cN/dtex, which are 122.2% and 30.6%, respectively, higher than the fibers treated in alkaline aqueous solutions. Such characteristics of high strength and high modulus are consistent with the hypothesis in Figure 4.

### 3.3. Penetration of Alkali in the Cotton Stalk Fibers

**Figure 6:**
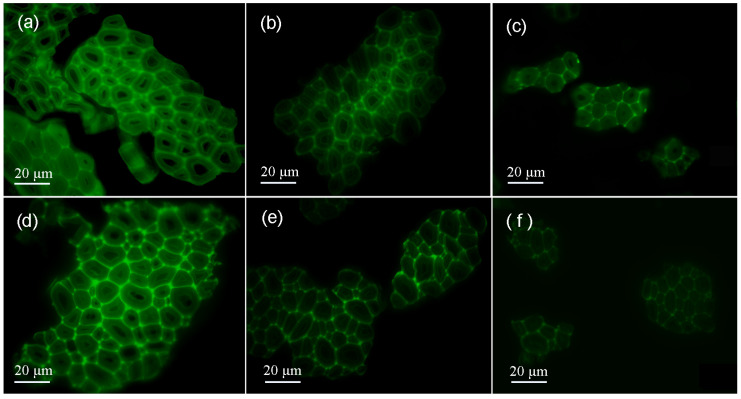
Cross section of the cotton stalk fibers treated in the alkali/glycerin mixtures (**a**) at the initial stage, (**b**) in the middle stage and (**c**) at the end of the treatment compared with that of the fibers treated in the alkaline aqueous solution (**d**) at the initial stage, (**e**) in the middle stage and (**f**) at the end of the treatment.

Figure 6 shows the cross section of the cotton stalk fibers treated in the alkali/glycerin mixtures compared with that of the fibers treated in the alkaline aqueous solutions. As seen in Figure 6a,d, the fibers treated at the initial stage have single cells, all with a distinct contour. In each cell, the intercellular layer has much brighter color than the wall, indicating that the former has a lignin concentration much higher than the latter. 

After a certain time of alkaline treatments in the glycerin solvents, the outer layer cells in the fibers turn dull, whereas the inner layer cells maintain their brightness (seen in Figure 6b). However, in the fibers treated in the aqueous phase (Figure 6e), the inner layer and outer layer are found both darkened. Differences between the two samples can be attributed to the different lignin removal ways. In the solvent of glycerin, the penetration of alkali is limited, and thus, only lignin in the outer layer can be removed. In the aqueous solutions, alkali penetrates quickly into the inner part. In such circumstances, lignin is removed from the inner layer and outer layer simultaneously. Since the secondary walls have a high ratio of S-type and non-condensation units, lignin in the walls is easier to remove than in the intercellular layers [21,22]. Thus, a significant brightness difference is observed between the walls and the intercellular layers (Figure 6e).

As seen in Figure 6c, the cotton stalk fibers that are obtained after the treatment in alkali/glycerin solutions have cross sections that remain bright. This phenomenon suggests that the inner structure of the fibers is unchanged. However, in the fibers that are treated in the aqueous phase, all cells almost lost their contours (seen in Figure 6f). Changes in the fibers cross section agree well with the hypothesis shown in Figure 4. 

**Figure 7:**
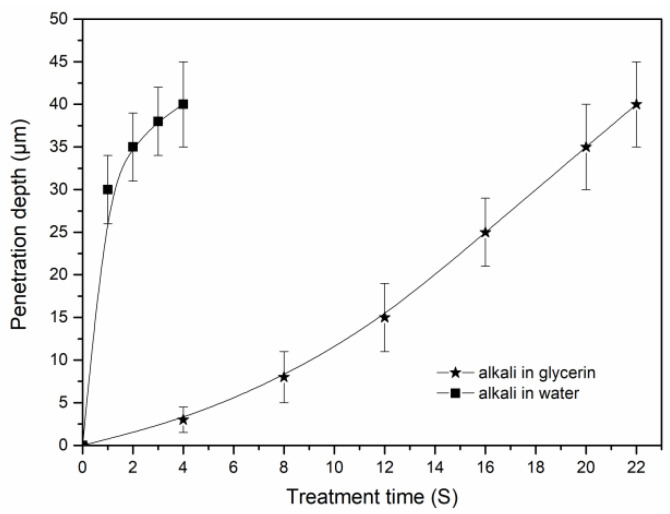
Penetration of alkali into the cotton stalk fibers within the glycerin and water solvents. The average diameter of the raw fibers is about 80 µm.

Figure 7 shows the penetration of alkali into the cotton stalk fibers within the glycerin and water solvents. As seen in Figure 7, the penetration of alkali in the water is much faster than that in the glycerin solvent. In the water phase, it requires only 3 to 4 s for alkali to reach the core site of the fiber bundles. However, the corresponding time in the glycerin solvent reaches 22 s. As seen from the figure, the penetration rate of alkali in the glycerin solvents shows an accelerated upward trend at initial time from 0 to 12 s. This phenomenon is highly consistent with the rapid reduction in the solution viscosity within the same time (seen in Figure 2b), confirming that the alkali penetration is mainly affected by the solvent viscosity. In this study, alkali penetration is limited, and the inner structure of the fibers remains intact. In such circumstances, the penetration rate of alkali is mainly determined by the thermodynamic movement of the solvents. Therefore, the alkali penetration may accelerate with the decrease in solvent viscosity.

### 3.4. Composition, Crystalline Structure and Thermal Stability of the Cotton Stalk Fibers

Table 2 shows the composition of the cotton stalk fibers treated in the solvent of glycerol and water. As seen from the table, the fibers treated in the glycerol solvent have a higher content of hemicellulose and lignin compared to the fibers treated in the water phase. In the water, alkali penetrates easily into the inner part of the fiber bundles. Thus, a simultaneous degumming phenomenon occurs between the inside and outside of the fiber bundles, resulting in fibers with relatively low hemicellulose and lignin content. In this study, only gum substances in the outer layer are removed. Thus, no significant changes were found in the composition of the fibers after the treatment in the glycerol solvents. This phenomenon agrees well with the model shown in Figure 4.

**Figure 8:**
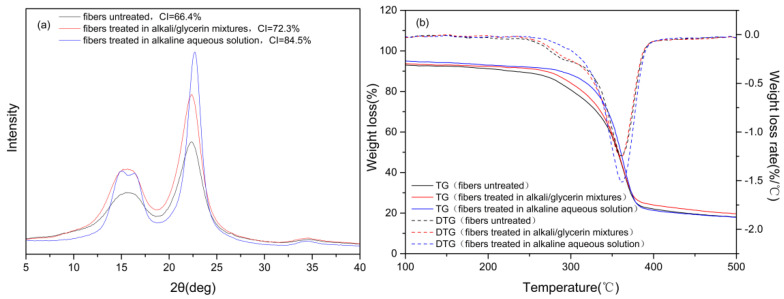
(**a**) X-ray diffraction intensity and (**b**) TG/DTG curves of the cotton stalk fibers treated in the alkali/glycerin mixtures compared with those of the fibers treated in the alkaline aqueous solution.

Figure 8a shows the X-ray diffraction intensity patterns of the fibers treated in the alkali/glycerin mixtures compared with those of the fibers treated in the alkaline aqueous solution. As seen in Figure 8a, all fibers have typical characteristics of cellulose Iβ. According to the report of French [23], peaks at 2θ angles of 14.9° and 16.6° correspond to the (1–10) and (110) lattice planes, respectively. These two peaks tend to merge into one broad peak in the untreated fibers, due to the presence of a high proportion of non-cellulose substances [3]. This phenomenon can be observed in the fibers treated in the glycerin solvent as well, suggesting that the gum substances are not substantially removed. However, a distinct difference is found in the fibers treated in the alkaline aqueous solution. Firstly, the peaks at 2θ angles of 14.9° and 16.6° are separated distinctly. Secondly, the peak at the 2θ angle of 22.6°, corresponding to (200) lattice planes, is found much sharper than the other two samples. All these characteristics suggest that non-cellulose substances are removed sufficiently from the fibers treated in the aqueous phase. The results of CI also confirmed this conclusion. As seen in Figure 8a, the treatment in the alkali/glycerin solution causes an 8.9% increase in the CI value, far lower than the 27.3% increase due to the treatment in the aqueous phase. In this study, alkali is limited in the outer layer and thus causes few changes to the inner structure of the fiber bundles.

Figure 8b shows the TG/DTG curves of the fibers treated in the alkali/glycerin solution compared with those of the fibers treated in the alkaline aqueous solution. As seen from the figure, the fibers treated in the glycerin solvent have DTG curves similar to the untreated fibers but distinctly different from the fibers treated in the aqueous phase. Firstly, the shoulder peak at 290 °C, corresponding to the decomposition of hemicellulose [24], is clearly seen in the fibers treated in the glycerin solvent but is almost absent in the fibers treated in the aqueous solution. Moreover, the main peak at 365 °C, corresponding to the decomposition of cellulose [24,25], is much blunter in the fibers treated in the glycerin solvent than in the fibers treated in the aqueous phase. All these characteristics confirm again that the inner structure of the fibers remains intact after the treatment in the alkali/glycerin solution.

### 3.5. Surface Morphology and Wettability of the Cotton Stalk Fibers

**Figure 9:**
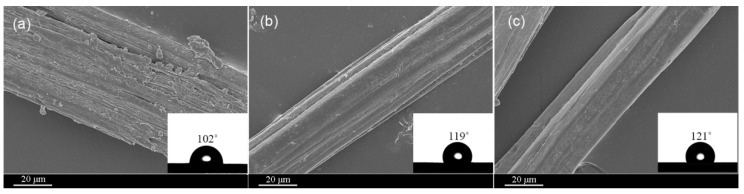
Surface morphology and water contact angle of the cotton stalk fibers (**a**) untreated, (**b**) treated in alkali/glycerin solution and (**c**) treated in alkaline aqueous solution.

Figure 9 shows the surface of the cotton stalk fibers treated in the glycerin solvents compared with that of the fibers treated in the aqueous phase. As seen in Figure 9a, the untreated fibers are coarse in diameter and covered with lots of gum on the surface. These gum substances are both substantially removed from the fiber surface after the treatment in the glycerin (Figure 9b) and aqueous solvents (Figure 9c), resulting in fibers with a clean surface. As seen from the figure, the treatment in the glycerin solvent causes a 16.7% increase in the water contact angle, slightly lower than the 18.6% increase in the alkaline aqueous solution. In this study, the accelerated temperature rise by means of microwave heating ensured an efficient removal of gum from the fiber surface. 

**Figure 10:**
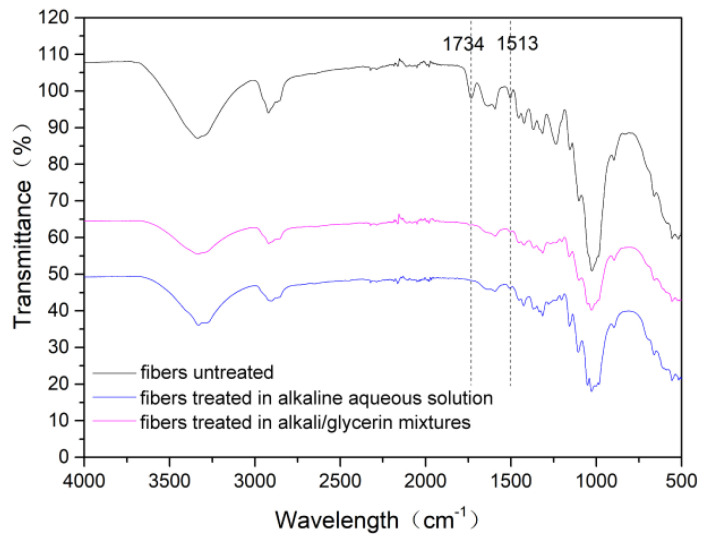
FTIR-ATR spectra of the fibers treated in the alkali/glycerin solution compared with those of the fibers untreated, as well as the fibers treated in the alkaline aqueous solution.

Figure 10 shows the FTIR-ATR spectra of the cotton stalk fibers treated under different conditions. As seen in Figure 9, the fibers treated in the glycerin solvents have infrared spectrum curves similar with the fibers treated in the aqueous phase but remarkably different from the fibers untreated. Firstly, the peaks at 1734 cm^−1^ are prominent in the untreated fibers but almost disappear in the other two samples. This peak corresponds to the C=O stretch vibration from hemicellulose and pectin [25]. Therefore, it can be concluded that hemicellulose has been sufficiently removed from the surface of the fibers treated in the glycerin solvent. Moreover, the peaks at 1513 cm^−1^, corresponding to the vibration of aromatic skeletal in lignin, are sharp in the untreated fibers but are significantly weakened in the fibers treated in the glycerin solvent. Lignin macromolecules have complex network structures and thus are difficult to be completely degraded within a few seconds, even as the temperature rises to 170 °C. In this study, most hemicellulose and partial lignin are removed from the fiber surface. This is a major reason for the significant increase in the water contact angle.

## 4. Conclusions

A combination of glycerin solvents and microwave-assisted heating technology is shown to be effective for extracting high-quality natural fibers from the bark of cotton stalks, due to a limited alkali penetration and simultaneous accelerated temperature rise. High power conditions result in an efficient stripping of single cells within a short time but cause a rapid decrease in the solution viscosity. An 8 wt % alkali concentration at 900 W for 22 s with a liquid to fiber ratio of 2:1 provided fibers with a fineness of 23.8 dtex, which is close to the best value reported in literature. The fibers, meanwhile, exhibit a high tensile strength at a break of 4.4 cN/dex and a Young’s modulus of 162.2 cN/dtex, which are 122.2% and 30.6%, respectively, higher than the fibers treated in the alkaline aqueous solution. Fluorescence microscope images of the fibers showed that alkali was limited in the outer layer of the fiber bundles during the treatment in the glycerin solvent. The results of XRD/DTG analysis confirm that the inner structure of the fibers retained intact after the treatment in the glycerin solvent. Moreover, the fibers also exhibit a clean surface and a 16.7% increase in the water contact angle. This work provides a new strategy for extracting high-quality lignocellulosic fibers that constitute a suitable candidate for potential reinforcing applications.

## Figures and Tables

**Table 1:** Experimental parameters for extracting cotton stalk fibers.

	Experimental Parameters		
	NaOH Concentration (wt %)	Microwave Power (W)	Microwave Time (S)
Range of values	2, 4, 6, 8	160, 480, 700, 900	0–300

**Table 2:** Composition of the cotton stalk fibers treated in the solvent of glycerol and water.

Cotton Stalk Fibers	Hemicellulose Content (%)	Lignin Content (%)	Cellulose Content (%)
Untreated	17.0 ± 2.1	24.6 ± 2.0	48.5 ± 3.2
In alkali/glycerol solution	16.8 ± 1.2	24.2 ± 1.7	50.8 ± 4.5
In alkaline water solution	4.6 ± 0.2	13.4 ± 0.7	78.4 ± 4.2

## Data Availability

The data supporting the reported results can be sent by email.
